# Majorbio Cloud: A one‐stop, comprehensive bioinformatic platform for multiomics analyses

**DOI:** 10.1002/imt2.12

**Published:** 2022-03-16

**Authors:** Yi Ren, Guo Yu, Caiping Shi, Linmeng Liu, Quan Guo, Chang Han, Dan Zhang, Lei Zhang, Binxu Liu, Hao Gao, Jing Zeng, Yong Zhou, Yuhan Qiu, Jian Wei, Yanchun Luo, Fengjuan Zhu, Xiaojie Li, Qin Wu, Bing Li, Wenyao Fu, Yanli Tong, Jie Meng, Yahong Fang, Jie Dong, Yitong Feng, Shichang Xie, Qianqian Yang, Hui Yang, Yan Wang, Junbiao Zhang, Haidong Gu, Hongdong Xuan, Guanqing Zou, Chun Luo, Long Huang, Bing Yang, Yachen Dong, Jianhua Zhao, Jichen Han, Xianglin Zhang, Huasheng Huang

**Affiliations:** ^1^ Shanghai Majorbio Bio‐pharm Technology Co., Ltd. Shanghai China

## Abstract

The platform consists of three modules, which are pre‐configured bioinformatic pipelines, cloud toolsets, and online omics' courses. The pre‐configured bioinformatic pipelines not only combine analytic tools for metagenomics, genomes, transcriptome, proteomics and metabolomics, but also provide users with powerful and convenient interactive analysis reports, which allow them to analyze and mine data independently. As a useful supplement to the bioinformatics pipelines, a wide range of cloud toolsets can further meet the needs of users for daily biological data processing, statistics, and visualization. The rich online courses of multi‐omics also provide a state‐of‐art platform to researchers in interactive communication and knowledge sharing.

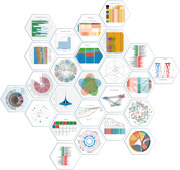

The rapid developments of high‐throughput sequencing technology in the last decade allowed the emergence of multiomics analyses. Analytic platforms for high‐throughput omics data, such as MG‐RAST [1], IMG/M [2], Qiita [3], BIGSdb [4], TRAPR [5], imageGP [6], and MetOrigin [7], have also emerged. Most of these platforms are designed for data from a single type of omics, especially metagenomics and transcriptomics. Also, many platforms are designed to be specialties for special problems instead of generalists that offer comprehensive solutions. For example, Metascape [8] is designed to only provide functional annotations of genes as well as function enrichment analysis, BioNumerics [9] and Ridom SeqSphere+ [10] perform multilocus sequence typing, while CARD [11] provides only antimicrobial resistance annotations. However, considering the complex structural system of living organisms, single omics analysis is insufficient in demonstrating the phenotype of organisms [12, 13, 14]. Thus, it is urgent to integrate multiple omics data together to facilitate life science researchers to identify new organisms, gene functions, metabolic, and regulatory networks.

Here we present the overall framework and each individual module of the Majorbio Cloud platform (https://cloud.majorbio.com/), which is a one‐stop, online analytic platform for high‐throughput omics data. The platform consists of three modules, which are preconfigured bioinformatic pipelines, cloud toolsets, and online omics' courses. The preconfigured bioinformatic pipelines not only combine analytic tools for metagenomics, genomes, transcriptome, proteomics, and metabolomics but also provide users with powerful and convenient interactive analysis reports, which allow them to analyze and mine data independently. As a useful supplement to the bioinformatics pipelines, a wide range of cloud toolsets can further meet the needs of users for daily biological data processing, statistics, and visualization. The rich online courses of multiomics also provide a state‐of‐the‐art platform for researchers in interactive communication and knowledge sharing. The Majorbio Cloud platform was released on October 26, 2016, and ever since has been used by 70,000+ researchers from 3562 institutes. It demonstrates that a one‐stop, comprehensive online platform can facilitate the use of multiomics data in all fields of biological analysis.

## CONVENIENT WORKFLOWS

**Figure 1:**
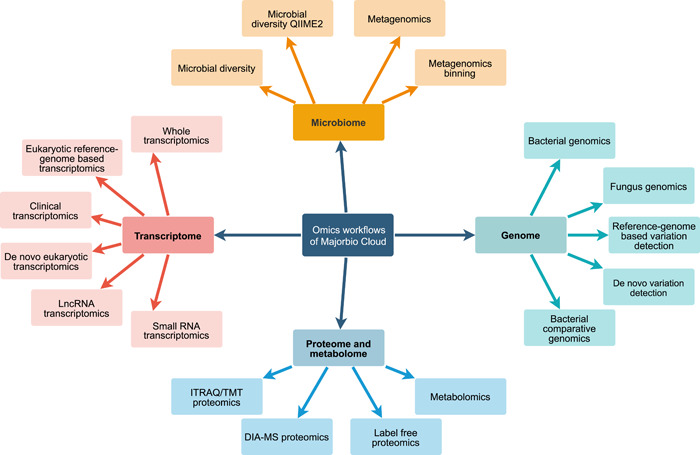
Tree map of omics workflows

We provide a visual web interface for each workflow to facilitate users to modify program parameters and customize the analysis process. Besides, Majorbio Cloud uses a self‐developed distributed task management system to implement the execution and monitoring of each workflow. In large‐scale computing clusters, the distributed task management system can accomplish the delivery of computing tasks, tracking of running state, and management of computing resources at the same time. At present, the Majorbio Cloud includes 18 bioinformatics workflows, such as microbiome, genome, transcriptome, proteome, and metabolome (see Figure 1 for details).

We also provide comprehensive documentation and video tutorials for each cloud workflow to help users easily implement omics data analysis in a one‐stop manner.

By reviewing the latest literatures and reviews, combined with our own experience in using various algorithms, Majorbio Cloud has built a flexible and variable omics analysis workflow for various applications. For each analysis module in the workflow, we provide a variety of algorithms, software, and databases, which users can choose independently according to the sampling background of sequencing data and their own preferences. Below we list two commonly used workflows, reference‐genome‐based eukaryotic transcriptomics and metagenomics, as examples. The tools used in reference‐genome‐based eukaryotic transcriptomics and metagenomics workflows are listed in Table S1.

**Figure 2:**
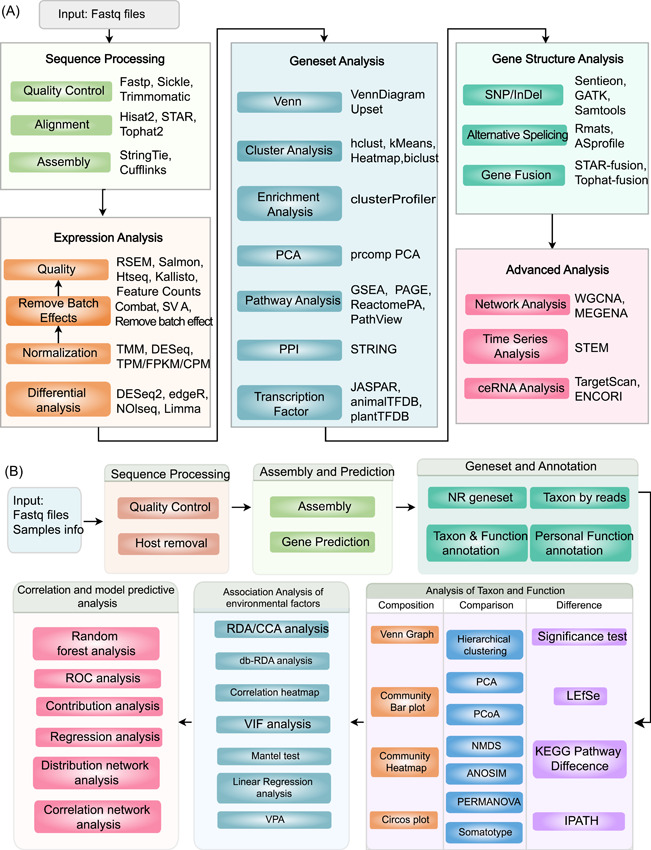
Examples of analysis workflows for Majorbio Cloud. (A) The eukaryotic transcriptome analysis workflow. The workflow contains five modules, fastq files run through processing, expression analysis, gene set analysis, gene structure analysis, and advance analysis. (B) The metagenome analysis workflow. The workflow is composed of six modules, fastq files and samples info run through processing, assembly and prediction, gene set and annotation, taxonomic and functional analysis, environmental factors analysis, association analysis, correlation, and model predictive analysis

The eukaryotic transcriptome analysis workflow (Figure 2A) is composed of five steps: data preprocessing, gene expression analysis, gene set analysis, gene structure analysis, and advanced analysis. In addition to conventional basic analysis, we also provide targeted analysis modules according to different research interests of users. For example, we provide a batch effect processing module to remove batch effects caused by RNA extraction, library preparation, RNA sequencing, and so forth. For disease and tumor research, single nucleotide olymorphisms calling, alternative splicing, and gene fusion analysis modules are supplied; we also offer time‐series expression trend analysis and time‐series differential expression analysis if the sequencing data are related to different development stages or different time points; moreover, WGCNA [15], MEGENA [16], competing endogenous RNA analysis modules are designed for the joint data mining of multiomics data.

The metagenome analysis workflow (Figure 2B) is composed of six steps: data preprocessing, assembly, gene prediction, gene set construction annotation, analysis of Taxon and Function, association analysis of environmental factors, and correlation and model predictive analysis. In addition to some general analyses, advanced modules are provided to different users. MetaPhlAn [17] method is provided for taxon annotation of the human‐derived specimen. For researchers who focus on the disease model, a series of methods, such as random forest model, receiver operating characteristic curve, and somatotype are provided to construct a disease diagnosis model. For research containing complex metadata information, variance inflation factor analysis is provided to remove redundant factors, while variance partitioning analysis enables researchers to figure out the impact of each type of factor on the microecological environment. Besides, function prediction analyses including virulence, secretion system, pathogen–host interaction are of avail for users interested in bacterial toxin and drug resistance mechanisms.

## INTERACTIVE ANALYSIS REPORT

Majorbio Cloud integrates data storage technologies (e.g., MongoDB, Ceph, MySQL) and D3 visualization technology in the framework that enables the analysis results generated by workflow into an interactive analysis report via a web browser in real‐time. With the support of an interactive analysis report, users can reprocess the result to realize the deep mining of omics data. The interactive analysis report allows users to mine their omics data visually with parameters selection, gene screening, gene sets creation, tables filtering, uploading local files for association analysis, image modification, and analysis records management.

Take gene set analysis as an example, many genes were detected by next‐generation sequencing for its high‐throughput capacity in omics research. Researchers were often required to mine genes related to the phenotype of the research target according to the information of gene expression, gene function, expression difference, or research background. For instance, users can arrange and combine different conditions, such as *p* value, adjusted *p* value, fold change (FC), log_2_FC threshold, genes with significant differences between groups, and upregulation or downregulation of expression in the differential expression analysis module to generate a gene set. The created gene set can be used for further screening in other modules, and users can also directly enter the gene set analysis module for multidimensional data mining and data visualization analysis, such as Venn analysis [18], cluster analysis, functional analysis, correlation analysis and enrichment analysis of COG/GO/KEGG [19]/DO/REACTOME [20], and so forth.

## ASSEMBLAGE OF BIOINFORMATIC ANALYSIS TOOLS

Currently, more than 300 analysis tools were integrated on Majorbio Cloud. These tools cover the classic software and methods in omics data analysis, including but not limited to sequence quality control, alignment, assembly, quantification, normalization, transformation, differential expression, dimensionality reduction, classification, clustering, variants, annotation, visualization, simulation, and imputation (Table S2). Majorbio analysis tools support multiple formats of data as inputs (e.g., fq, txt, csv, bam). A friendly graphical user interface is developed to help users manipulate data easily. Either file uploaded from users or output by bioinformatic pipelines is allowed as inputs for tools. Then, personalized data analyses could be carried out by setting the analysis parameters simply and quickly. All the result files can be downloaded, and moreover, graphical outputs can be downloaded as either portable document format or scalable vector graphics files for publication purposes. Besides, tutorials in the form of documents or videos are prepared for each tool. Therefore, Majorbio bioinformatic analysis tools provide users with a useful supplement to workflow and interactive reporting, which makes personalized data mining more flexible and efficient.

## USER‐FRIENDLY INTERACTIVE CHARTING AND VISUALIZATION SANGER‐CHARTS

**Figure 3:**
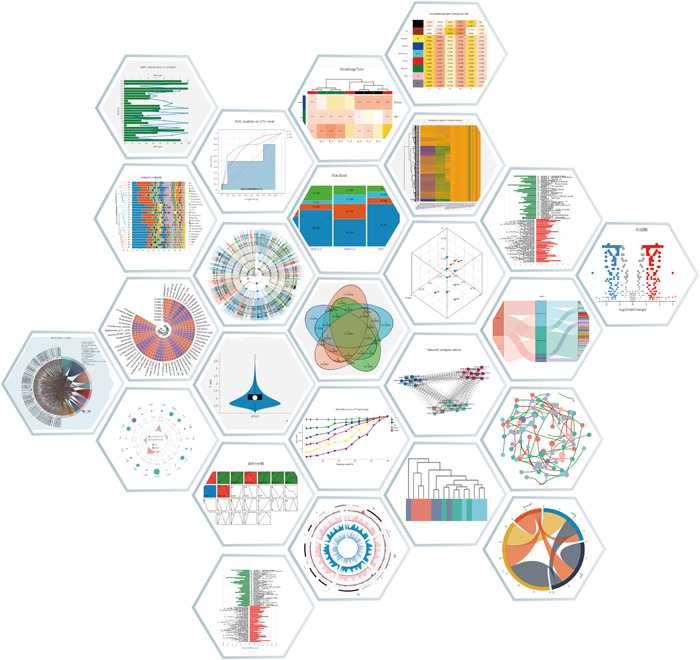
Example of Sanger‐Charts

Majorbio Cloud uses the self‐developed interactive charting and visualization Sanger‐Charts. Sanger‐Charts provides a wealth of chart types and display examples (Figure 3). In addition to conventional charts in the field of data statistics and analysis, it also has customized designs for the characteristics of many types, formats, and large data sets in the life sciences field. The flexible parameter configuration of Sanger‐Charts supports multiple combinations of graphic elements and interactive real‐time chart presentation. Sanger‐Charts also has a self‐adaptive charting function and modification record saving function. Sanger‐Charts, a highlighted part of Majorbio Cloud, has been widely used and recognized by users.

## ONLINE OMICS COURSES

Majorbio Cloud builds a well‐established omics knowledge sharing and interactive communication platform for users. Cloud Classroom regularly releases live training of omics courses to help users systematically learn omics‐related knowledge concepts and analysis methods. Cloud Library brings together the latest and most cutting‐edge research trends in the field of omics. At present, experts and scholars from universities, scientific research institutes, and technical engineers of Majorbio have shared 3795 learning videos on the Majorbio Cloud, involving research progress, experimental techniques, technical principles, code practice, and so forth. Online classrooms (http://edu.majorbio.com/) have been serving more than 60,000 users and accumulatively playing more than one million times.

## USERS AND PUBLICATIONS

Focusing on providing omics bioinformatic analysis services, since October 2016, the services content of the platform has been upgraded and iterated over 100 times. There are already more than 70,000 scientific research users, involving over 3000 well‐known universities and institutes, who have totally completed more than 300,000 omics research standardized data analysis and data mining tasks. In the past 4 years, over 1200 journal articles cited Majorbio Cloud in their methods.

## CONFLICTS OF INTEREST

The platform described in this manuscript is related to an authorized patent CN201810796979.6. Quan Guo, Guo Yu, Yi Ren, Lei Zhang, Yong Zhou, Xianglin Zhang, and Huasheng Huang are inventors of the patent. Huasheng Huang and Xianglin Zhang are cofounders of Majorbio. The other authors are employees of Majorbio.

## AUTHOR CONTRIBUTIONS

Yi Ren conceived the platform and idea. ChangHan, Dan Zhang, Guo Yu, Linmeng Liu, and Caiping Shi wrote the manuscript. Chang Han was responsible for editing and revising the manuscript. All authors contributed to the development of Majorbio Cloud.

## Supporting information

Supporting information.

Supporting information.

## Data Availability

Supporting Information tables include tools list of reference‐genome‐based eukaryotic transcriptome analysis and metagenome analysis workflow, which are available online.
