# Supplementary material for: Majorbio Cloud: A one‐stop, comprehensive bioinformatic platform for multiomics analyses
Source: Imeta. 2022 Mar 16;1(2):e12. doi: 10.1002/imt2.12 (PMC10989754; doi:10.1002/imt2.12)
Supplement: Supplementary file 2 — Supporting information. [file IMT2-1-e12-s002.docx]

SUPPORTING INFORMATION

Additional supporting information (supplementary tables) may be found in the online version of the article at the publisher’s website.
